# ENAP1 retrains seed germination via H3K9 acetylation mediated positive feedback regulation of *ABI5*

**DOI:** 10.1371/journal.pgen.1009955

**Published:** 2021-12-15

**Authors:** Bo Zhao, Likai Wang, Zhengyao Shao, Kevin Chin, Daveraj Chakravarty, Hong Qiao

**Affiliations:** 1 Department of Molecular Biosciences, The University of Texas at Austin, Austin, Texas, United States of America; 2 Institute for Cellular and Molecular Biology, The University of Texas at Austin, Austin, Texas, United States of America; Peking University, CHINA

## Abstract

Histone acetylation is involved in the regulation of seed germination. The transcription factor ABI5 plays an essential role in ABA- inhibited seed germination. However, the molecular mechanism of how ABI5 and histone acetylation coordinate to regulate gene expression during seed germination is still ambiguous. Here, we show that ENAP1 interacts with ABI5 and they co-bind to ABA responsive genes including *ABI5* itself. The hypersensitivity to ABA of *ENAP1ox* seeds germination is recovered by the *abi5* null mutation. ABA enhances H3K9Ac enrichment in the promoter regions as well as the transcription of target genes co-bound by ENAP1 and ABI5, which requires both ENAP1 and ABI5. *ABI5* gene is directly regulated by ENAP1 and ABI5. In the *enap1* deficient mutant, H3K9Ac enrichment and the binding activity of ABI5 in its own promoter region, along with ABI5 transcription and protein levels are all reduced; while in the *abi5-1* mutant, the H3K9Ac enrichment and ENAP1 binding activity in *ABI5* promoter are decreased, suggesting that ENAP1 and ABI5 function together to regulate ABI5- mediated positive feedback regulation. Overall, our research reveals a new molecular mechanism by which ENAP1 regulates H3K9 acetylation and mediates the positive feedback regulation of ABI5 to inhibit seed germination.

## Summary

To optimize the fitness in natural environment, flowering plants initiate seed germination in the favorable environment and maintain seed dormancy under stressful conditions. Precise mechanisms have been evolved to regulate germination timing to ensure plant adaptation to unfavorable environment. ABA, a major stress hormone in plants, induces seed dormancy and represses seed germination. Epigenetic regulation has been known involved in ABA signaling in which the transcription factor ABI5 acts as a regulatory hub. However, the epigenetic regulation such as histone acetylation on *ABI5* transcription remains elusive. In this study, we revealed a new molecular mechanism by which histone binding protein ENAP1 regulates H3K9 acetylation, which mediates the positive feedback regulation of ABI5 in an ABI5 dependent manner to inhibit seed germination.

## Introduction

Seed germination is critical in the life of a plant as it determines success of future growth and development. Seed germination commences with the absorption of water by dry seeds and ends with the elongation of the embryonic axis and emergence of radicle. The process is delicately regulated to maximize the plant’s survival in response to various environmental stimuli [1,2]. The plant hormone abscisic acid (ABA) is a primary regulator of seed dormancy and germination as well as to respond to abiotic stresses in many plant species. ABA signaling is implemented by a number of AREB/ABF (ABA Responsive Elements- Binding Factors) transcription factors (TFs) including bZIP family proteins that bind to AREB (ABA Responsive Elements) to regulate downstream gene expression [3–5].

The bZIP transcription factor ABI5 plays a critical role in the regulation of ABA- mediated seed germination and early seedlings growth. The *abi5* mutant was first characterized as ABA insensitive during seed germination [6,7]. The expression of *ABI5* is induced by ABA within a short developmental window post germination, during which plants evaluate environmental conditions before initiating vegetative growth [8]. It’s hypothesized that ABI5 is necessary to bring germinated seeds to quiescent state under osmotic stress and thus protects the young seedlings from losing water [8]. To maintain the embryo in a balance of active and quiescent status in corresponding conditions, ABI5 is closely regulated in transcriptional and post-translational levels [9]. Multiple transcription factors and other proteins are involved in positive or negative regulation of *ABI5* transcription, such as positive regulators ABI3 and DOG1 [10,11], negative regulators MYB7 and RAV1 [12,13]. Interestingly, ABI5 is undergoing autoregulation by binding to its own promoter. The yeast expressing reporter construct of *pABI5*::*lacZ* and GAL4 activation domain-ABI5 fusion (GAL4AD-ABI5) shows much higher β-galactosidase activity than yeast only expressing GAL4 activation domain, indicating ABI5 can target its own promoter in *trans* [14]. Yeast one-hybrid provides further evidence that ABI5 directly activates its own expression [15]. In the presence of ABA, ABI5 is activated and prompted to bind to the promoters of a set of genes that contain AREB motifs [7,16]. The ABI5 binding motif is prominent in the promoter regions of certain *LEA* gene families that respond to ABA for seed desiccation tolerance establishment [17]. The expression of *ABI5* is correlated with the ABA- mediated desiccation tolerance re-establishment in germinated seeds [18].

Histone acetylation is reported to be involved in seed germination, and numbers of studies have revealed the critical role of histone deacetylase in this regard [19,20]. Histone deacetylases HDA6 and HDA19 act redundantly to repress embryonic genes expression after germination, with seed germination of *hda6* and *hda19* mutants being hypersensitive to ABA [21–23]. Further studies show that HDA19 interacts with SNL1 to regulate histone acetylation at H3K14/18Ac in seed dormancy [24]. HDA9, another histone deacetylase, acts together with PWR to deacetylate H3K9Ac and H3K14Ac at target genes, and their deficient mutants display faster seed germination [25,26]. More recent studies revealed that HDA15 participates in ABA signaling by interacting with transcription factor MYB96. In the presence of ABA, the HDA15-MYB96 complex co-binds to the promoters of a subset of *RHO GTPASE OF PLANTS (ROP)* genes and removes the acetyl groups of H3 and H4, consequently resulting in the repression of their expression. In support, the sensitivity to ABA during seed germination in the *hda15* and *myb96* loss-of-function mutants is reduced [27]. Additionally, other HDAC families are also reported to modulate seed germination. In the *hd2c* mutant, the elevation of H3K14Ac and reduction of H3K9me2 lead to the activation of *ABI1* and *ABI2*, resulting in an enhanced sensitivity to ABA during germination [28]; Similarly, the acetylated H3 is accumulated in *hdc1* mutant which displays a hypersensitive seed germination phenotype in the presence of ABA [29]. Given the essential role of ABI5 in ABA- inhibited seed germination, histone acetylation mediated regulation of *ABI5* is of great interest, yet the molecular mechanism is still largely unknown.

ENAP1 has a SANT domain at its N-terminus, and it’s first identified as the interacting protein of Agrobacterium VirF protein [30]. ENAP1 is a histone binding protein that regulates histone acetylation [31–34]. ENAP1 preferentially binds to regions associated with actively transcribed genes and creates a relative relax chromatin status for rapid response to stimulus such as ethylene [33]. In this study we demonstrate that the histone binding protein ENAP1 acts as a positive regulator in the ABA pathway to restrain seed germination. The *enap1* deficient mutants display reduced sensitivity to ABA during seed germination, which is opposed to the *ENAP1* gain-of-function (*ENAP1ox*) mutant that is hypersensitive to ABA during seed germination. We further find that ENAP1 interacts with ABI5 and they co-bind to ABA responsive genes including *ABI5* itself, and the *abi5* null mutation restores the hypersensitivity to ABA of *ENAP1ox* seed germination. Moreover, both ENAP1 and ABI5 are required for ABA to enhance H3K9Ac levels in the promoter regions of their co-targeted genes and thereby to regulate their expression. Finally, the decrease of H3K9Ac in the *ABI5* promoter and the ABI5 binding activity in the *enap1* deficient mutant leads to a reduction of *ABI5* transcription, showing the requirement of ENAP1 in H3K9Ac mediated positive feedback regulation of *ABI5*. These results reveal a new molecular mechanism by which ENAP1 regulates histone acetylation and mediates the positive feedback regulation of ABI5 to inhibit seed germination.

## Materials and methods

### Plant materials and growth conditions

**Fig 1:**
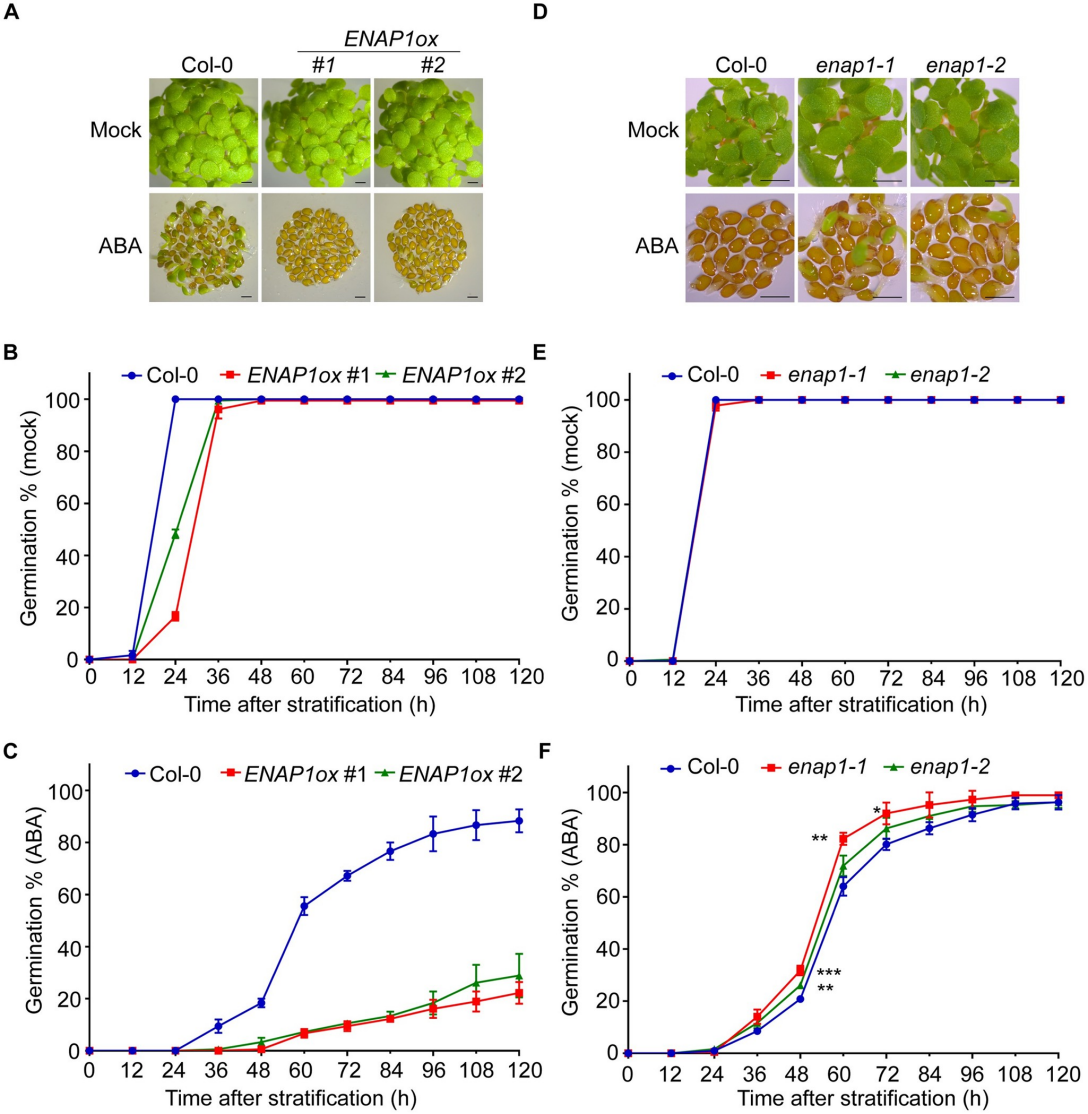
ENAP1 positively regulates ABA response to inhibit seed germination. (**A**) Germination phenotype of Col-0 and *ENAP1ox* independent lines. Stratified seeds of wild type (Col-0) and *ENAP1ox* (#1, #2) were germinated on ½ MS containing ethanol (mock) or 2 μM ABA. Photographs were taken at 96h after stratification. Bar, 0.1mm. (**B and C**) Quantification of seeds germination rates in Col-0 and *ENAP1ox* lines. Seeds were germinated on ½ MS with mock (**B**) and 2μM ABA (**C**) treatment. Germinated seeds (radicle emerged) were recorded every 12h until 120h after stratification. (**D**) Germination phenotype of Col-0 and *enap1* CRISPR/Cas9 deletion lines. Seeds germinated for 72h after stratification under mock (ethanol) or 2μM ABA treatment were imaged. Bar, 0.1mm. (**E and F**) Germination percentages of Col-0 and *enap1* deletion lines. Stratified seeds of Col-0, *enap1-1* and *enap1-2* were germinated on ½ MS supplemented with ethanol (**E**) and 2μM ABA (**F**). Germinated seeds were counted every 12h until 120h after stratification. All quantified data represent means ± s.d. of at least 180 seeds in three replicates. Seed germination rates of *enap1-1* and *enap1-2* at 48h, 60h and 72h were compared to Col-0 with unpaired two-tailed t-test. * *P* < 0.05; ** *P* < 0.01; *** *P* < 0.001.

All Arabidopsis plants used in this study are Col-0 background except the *abi5-1* plants which are in the Ws background. *35S*::*ENAP1*:*YFP*:*HA* (*ENAP1ox*) and *pENAP1*::*ENAP1-YFP*/Col-0 have been described before [31]. The *ENAP1ox* #1 was used for all other experiments except the seed germination assay in Fig 1. *ENAP1ox/abi5-1* was generated by crossing *ENAP1ox* #1 with *abi5-1*. Sanger sequencing confirmed *abi5-1* point mutation in *ENAP1ox/abi5-1*. Salk_200891, named *abi5-10*, is a T-DNA insertion line of *ABI5*, and it’s crossed with *pENAP1*::*ENAP1-YFP*/Col-0 to generate pENAP1::ENAP1-YFP/*abi5-10*. For *enap1 CRISPR* mutants, hygromycin-resistant T1 plants from Agrobacterium-mediated transformation were screened by PCR. Followingly, truncated *ENAP1* genomic DNA fragments were cloned to pBlunt for sequencing. Two homologous deletion mutants *enap1-1* (generated by gRNA1 + gRNA2) and *enap1-2* (generated by gRNA3 + gRNA4) were obtained in the T1 population. In the T2 generation, *Cas9*-free plants were identified by PCR of *Cas9* and further confirmed by hygromycin screening. Homologous and *Cas9*-free T3 plants were used for the experiment. Seeds were harvested from plants grown in the long day condition (16h light / 8h dark, 22°C). Seeds were after- ripened in a dry condition for at least one month before performing experiments.

Genes referenced in this article were found in the *Arabidopsis* Genome Initiative database with following accession numbers: *ENAP1*(At3G11100), *ABI5*(AT2G36270), *AtEM1*(At3g51810), *LEA4-1*(At3g17520), *REV3*(AT1G01520), *ABF1*(AT1G49720), *ABF2*(AT1G45249), *ABF3* (AT4G34000), *ABF4* (AT3G19290).

### Seed germination assay

Dry seeds were surface sterilized using 50% bleach with 0.01% (v/v) Triton X-100 for 7 mins and washed five times with sterile water. Then the sterilized seeds were placed on 1/2 MS medium plates containing 0.8% (w/v) Phytoblend Agar (Caisson Labs) supplemented with 0.01% (v/v) ethanol as mock or 2μM ABA (Sigma-Aldrich, dissolved in 100% ethanol) as treatment. In total, 180 seeds (60 per replicate) were used for each genotype. All plates with seeds were placed at 4°C in the dark for 3 days and then transferred to long day condition (16h light / 8h dark, 22°C) for further analysis. The germination event was defined as the emergence of the radicle, and germinated seeds were counted every 12 h until 120h after stratification.

### Plasmid construction

The CRISPR/Cas9 vector construction has been described before [35]. Briefly, potential gRNAs targeting ENAP1 were searched and evaluated in CRISPR-Plant [36]. A pair of guide RNA (gRNA) oligos were incorporated in gRNA transcription cassette from pDT1T2 by PCR and cloned to pHEE401E destination vector for Agrobacterium-mediated transformation. To construct vectors for yeast two-hybrid, the coding sequence (CDS) of *ABI5* and *ENAP1* were amplified and ligated to pDBLeu (Invitrogen) and pEXP-AD502 (Invitrogen) respectively, giving rise to BD-ABI5 and AD-ENAP1. CDS sequences of *ABF1*, *ABF2*, *ABF3* and *ABF4* were *cloned to* pEXP-AD502 to generate AD vectors. For the pull-down assay, *ABI5* and *ENAP1* CDS were ligated to pET28a and pVP13 (Gateway vector, His tag removed) to generate His-ABI5 and MBP-ENAP1. For the BiFC experiment, *ABI5* CDS was cloned to pDEST-VYNE, and *ENAP1* CDS was cloned to pDEST-VYCE. *ACT8* CDS was cloned to both BiFC destination vectors to serve as a control. All constructs above were verified by sequencing. Primers used are listed in the S1 Table.

### Western blots for ABI5 and ENAP1 proteins

Seeds were sterilized and stratified for 3 days at 4°C in the dark and placed on ½ MS plates containing ABA or 0.01% (v/v) ethanol (mock) to germinate for indicated times in the long day condition. Seeds for protein analysis were collected and quickly frozen in liquid N_2_ and stored in -80°C before processing. Seeds were ground in liquid N2 and dissolved with 2X protein loading buffer [50mM Tris-HCl (pH6.8), 2% SDS, 10% glycerol, 0.01% Bromophenol Blue, and freshly added 0.4% (v/v) β-mercaptoethanol]. Total proteins were resolved by SDS-PAGE, transferred to a PVDF membrane, then probed with anti-ABI5 antibody (Abcam) or anti-HA antibody (Cell Signaling). RPT5 or H3 was used as the loading control.

### Gene expression analysis

Total RNA was extracted using PureLink Plant RNA Reagent (Invitrogen). First cDNA strand was synthesized with a NEB ProtoScript II Reverse Transcriptase kit. qRT-PCR was performed by combining cDNA with SYBR master mix in the Roche thermos cycler. Each sample was analyzed in triplicate. Gene expression levels were normalized to UBQ10.

### Yeast two-hybrid assay

The yeast two-hybrid assay was performed following a previous published process by using the ProQuest Two-Hybrid System (Invitrogen) [34]. Briefly, AD and BD vectors fused with genes of interest were co-transformed into the yeast strain AH109 (Clontech). The transformants were grown on SD/-Trp-Leu or SD/-Trp-Leu-His dropout medium after sequential dilution. The growth of yeast on SD/-Trp-Leu-His selective medium supplemented with 3’AT (Fisher Scientific) indicated the interaction between proteins of interest.

### *In vitro* pull-down assay

The empty MBP (control) and MBP-ENAP1 proteins were purified from *E*.*coli* with amylose resin (NEB). After washing with column buffer (20 mM Tris-HCl pH 7.4, 0.2 M NaCl, 1 mM EDTA, 1mM PMSF, protease inhibitor) three times and eluted with column buffer containing 10mM maltose (Fisher Scientific). MBP fused protein were dialyzed with an amicon filter (EMD Millipore) and dissolved in the column buffer for further analysis. His-ABI5 was purified with Ni-NTA agarose (QIAGEN) and incubated with dialyzed MBP fused proteins for 1h at 4°C. After being washed five times with column buffer, proteins were collected by centrifuging the sample and were resuspended in column buffer for western blot analysis. Pull-down products were separated by SDS-PAGE and immunoblotted with anti-His (Sigma) and anti-MBP (NEB) antibodies.

### BiFC assay

Agrobacterium-infiltration was used for the transient expression of gene constructs in 4–6 week tobacco leaves [37]. Briefly, Agrobacterium transferred with plasmids were inoculated overnight at 28°C. Agrobacterium cells were collected by centrifuge and re-suspended in fresh infiltration buffer (10mM MES/KOH pH5.7, 10mM MgCl2, 100μM Acetosyringone). The different BiFC-partner strains as well as the p19 strain were diluted and mixed to yield a final OD600 of 0.4. The Agrobacterium mixture was slowly shaken in 28°C for 3h, and then filtrated onto tobacco leaves. Two days after infiltration, fluorescence of leaf discs was observed under the confocal microscopy (Zesis 710). DAPI was used to stain the nuclei.

### Co-immunoprecipitation assay

Crude proteins were extracted from *ENAP1ox* seeds germinated on ½ MS medium with 2 μM ABA for 24h after stratification with Co-IP buffer (50mM Tris–Cl pH 8.0,150mM NaCl, 1mM EDTA, 0.1% Triton X-100, 1mM PMSF and protease inhibitor). Anti-ABI5 antibodies were incubated with Dynabeads Protein G (Thermo) for 5h before being applied. Followingly, the anti-ABI5 and Dynabeads Protein G mixture were incubated with the crude protein solution overnight at 4°C. After washing five times, proteins bound on the Dynabeads were collected, and resuspended with 2X protein loading buffer. IP proteins were separated with SDS-PAGE and immunoblotted with anti-ABI5 (Abcam) and anti-HA (Cell Signaling) antibodies to detect ABI5 and ENAP1.

### ChIP assay

ChIP assays were performed as previously described [31]. Generally, seeds were collected and fixed with 1% formaldehyde. The chromatin was isolated and sonicated to generate DNA fragments with an average size between 300–500bp. Then solubilized chromatin was immunoprecipitated using Protein G Dynabeads (Thermo) and incubated with antibodies [anti-ABI5 (Abcam), anti-GFP (Invitrogen), anti-H3Ac (EMD Millipore), anti-H3K9Ac (EMD Millipore), anti-H3K14Ac (EMD Millipore), anti-H3K18Ac (EMD Millipore) and H3K27Ac (EMD Millipore)]. The co- immunoprecipitated DNA was recovered and analyzed by real-time PCR. All ChIP-qPCR primers used in this paper are listed in Supplemental Table. S1.

### RNA-seq and ChIP-seq analysis

RNA-seq analysis under ABA treatment and *ENAP1ox* RNA-seq analysis were described previously [31,38]. For the top 10 TFs expression change, raw counts from RNA-seq of seeds in different germination time [39] were converted to TPM (Transcripts Per Million) for plotting. RNA-seq raw data of *abi5-1* seeds germinated for 24h were obtained from GSE90004 [40], and analyzed with FastQC for quality control (bioinformatics.babraham.ac.uk/projects/fastqc/). Paired-end reads were mapped to the Arabidopsis genome (TAIR10) with botwie2 (2.4.2) with default parameters [41]. Mapped reads were counted by featureCounts (Subread 2.0.1) for each gene [42]. Differentially regulated genes were identified using DESeq2 (1.32.0) with a p-value <0.01, q-value <0.05 and | log2(fold change) | >1 [43]. For ABI5 and ENAP1 ChIP-seq analysis [31,44], raw sequencing data were obtained from the database Gene Expression Omnibus (GEO) and had quality control performed with FastQC (bioinformatics.babraham.ac.uk/projects/fastqc/). Low quality reads were removed with Trim Galore (0.6.7) (bioinformatics.babraham.ac.uk/projects/trim_galore/) and then mapped to the Arabidopsis genome using botwie2 (2.4.2) [41]. To show the ChIP signals surrounding TSS of each gene in certain category, genome-wide read coverage of merged bam files from two replicates were calculated with bamCompare (deepTools 3.5.1) using default parameters [45]. Each read coverage was normalized as RPKM relative to the input ChIP signal. ChIP signal scores per genome regions were calculated by computeMatrix (deepTools 3.5.1) and the mean of scores in each region was used to make the profile plot [45].

## Results

### ENAP1 positively regulates ABA response during seed germination

ENAP1 is a histone binding protein that mediates histone acetylation in the ethylene response [31]. During our research, we noticed that the seed germination of the gain-of-function of *ENAP1* (*ENAP1ox*) was slower than the wild type (Col-0). In line with our previous observation, the germination assay showed that the germination rates of *ENAP1ox* seeds were much lower than that of Col-0 (Fig 1A and 1B). In addition, *ENAP1ox* seed germination rates were negatively correlated with the *ENAP1* gene expression (S1A Fig). It has been well documented that ABA plays a significant role in seed germination [2]. To explore whether ENAP1 is involved in ABA-regulated seed germination, we compared seed germination rates of *ENAP1ox* and Col-0 in the presence of 2μM ABA. Compared to Col-0, the *ENAP1ox* seeds germination was much more sensitive to ABA treatment (Fig 1A and 1C). Consistently, *ENAP1ox* seeds exhibited higher sensitivity to ABA than Col-0 under other concentrations of ABA treatment (S1B Fig).

To further examine the function of ENAP1 in seed germination, we first tested the seed germination in the *ENAP1* knocking- down lines (*amiR-ENAP1*) with or without ABA treatments (S1C Fig). In the absence of ABA, no difference was observed in the seed germination between Col-0 and *amiR-ENAP1* lines (S1D Fig). In the presence of ABA, the *amiR-ENAP1* lines showed less sensitivity to ABA compared to Col-0 during seed germination, suggesting that ENAP1 negatively regulates seed germination via ABA pathway (S1E Fig). To further confirm this result, we generated *enap1* mutants by CRISPR/Cas9. We obtained two independent lines, *enap1-1* that carried a 146bp deletion and *enap1-2* that carried a 30bp deletion, both of which introduced an early stop to ENAP1 translation and resulted in 120 a.a and 61 a.a truncations from the C- terminus (S1F–S1I Fig). RT-PCR showed that the expression of truncated *ENAP1* was largely decreased in both lines (S1J Fig). In the absence of ABA, no difference was observed in the seed germination of *enap1-1* and *enap1-2* compared to Col-0 (Fig 1D and 1E). In the presence of ABA, both *enap1-1* and *enap1-2* seeds germinated faster than Col-0 (Fig 1D and 1F). More interestingly, the *enap1-1*, which harbored a longer protein truncation, displayed a less sensitivity to ABA in seed germination than *enap1-2*, showing that the longer truncation in ENAP1 leads to a more sever phenotype (Fig 1F, S1F and S1G Fig). Together, these data suggest that ENAP1 inhibits seed germination via ABA.

### ENAP1 is involved in the regulation of ABA responsive genes

**Fig 2:**
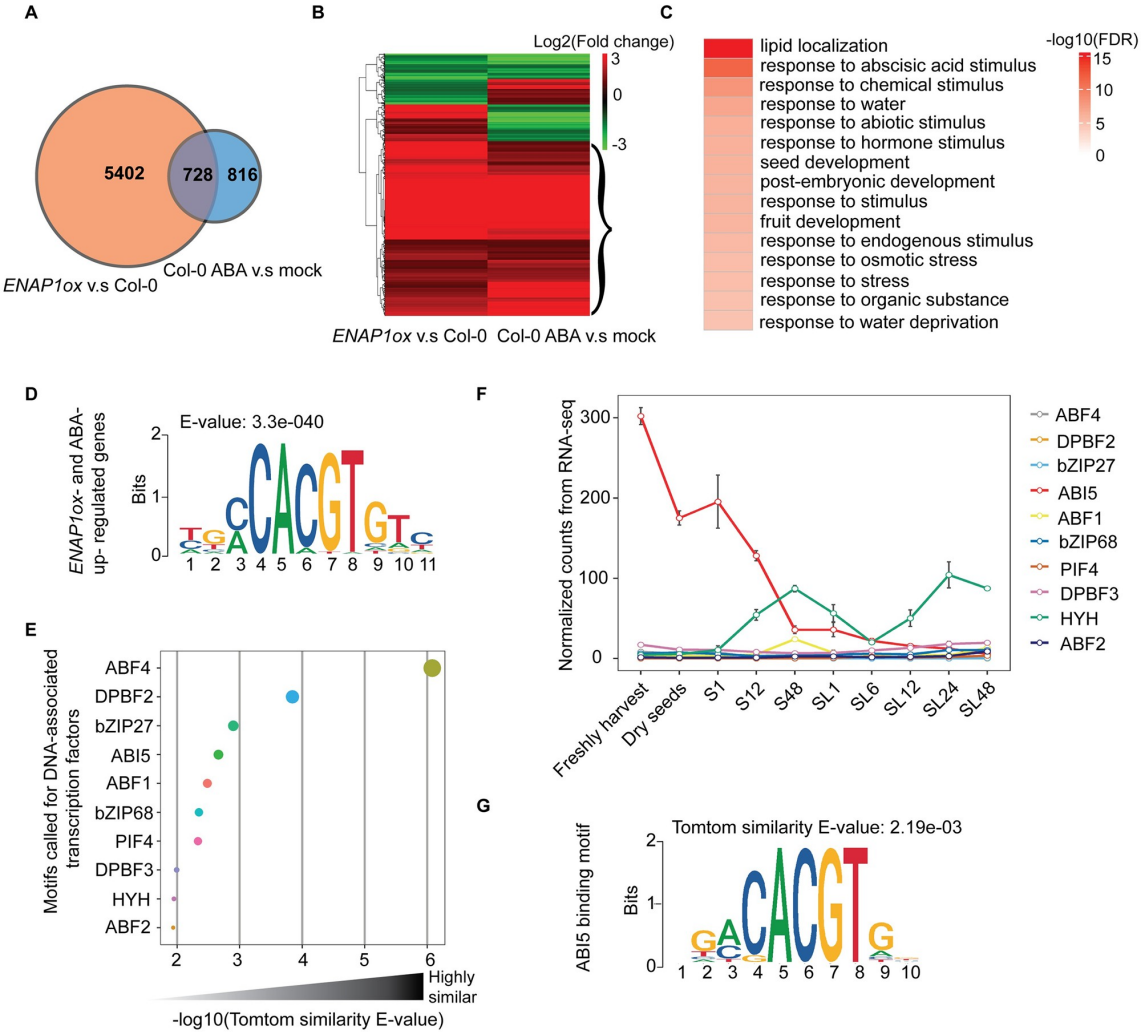
ENAP1 is involved in the regulation of ABA-responsive genes. (**A**) Venn diagram showing the number of genes regulated by ABA and ENAP1. (**B**) Heatmap to show the correlation of genes differentially regulated by ABA and ENAP1. Curly bracket indicates genes both up- regulated by ABA and ENAP1 (totally 485 genes). (**C**) Gene Ontology analysis of the genes that are both up- regulated by ABA and ENAP1. (**D**) DNA motif that was identified from 1k bp upstream of TSS in ABA and ENAP1 up- regulated genes. (**E**) The motif in (**D**) was compared to all plant motifs in CIS-BP2.0 database with Tomtom motif comparison tool [54], and top 10 motifs were selected. TFs associated with those motifs were plotted according to similarity scores (-log10(E-value)). (**F**) Expression levels of top 10 TFs during seed germination. Raw counts from published RNA-seq data [39] were normalized to the TPM (Transcripts Per Million). Samples were from freshly harvested and dry seeds, and seeds following indicated time of stratification (S) and post-stratification (SL). Each data point represents mean ± s.d. of three replicates. (**G**) ABI5 binding motif from CIS-BP2.0 database. Tomtom E-value indicates the similarity between ABI5 binding motif and YGMCACGTGTC motif.

To explore how ENAP1 is involved in ABA pathway, we revisited previously collected *EANP1ox* RNA-seq data, and compared the differentially expressed genes in *EANP1ox* with the genes that are differentially regulated by ABA [31,38]. We found that about 47% (728/1544) of ABA responsive genes were also differentially regulated in *ENAP1ox* plants (Fig 2A). More importantly, the expression patterns of those co-regulated genes were highly correlated, and about 67% (485/728) were both positively modulated by ENAP1 and ABA (indicated by curly bracket in Fig 2B). Gene Ontology (GO) analysis on these 485 genes revealed that the term “response to ABA stimulus” was significantly enriched (Fig 2C), showing that ENAP1 is involved ABA response.

ENAP1 was reported to mediate ethylene response through its association with ethylene responsive transcription factor (TFs) [31]. ENAP1 was not significantly regulated by ABA in the protein level (S2A Fig), and hence there might be ABA responsive TFs associated with ENAP1 to respond to ABA, mimicking the scenario in the ethylene response. To identify potential transcription factors involved in ENAP1-ABA regulation module, we extracted 1 kb upstream DNA sequence of the TSS (transcription starting site) in ABA and ENAP1 co-upregulated genes and searched the conserved motifs using MEME motif discovery software [46]. The motif YGMCACGTGTC was highlighted with an E-value 3.3e-40 (Fig 2D). This motif was then matched with the TF binding sites database CIS-BP2.0 to find similar motifs and the associated TFs [47]. In total, 67 motifs were discovered with a p-value cutoff 0.01, and AREB binding motifs appeared in the top 10 motifs (Fig 2E). To further pinpoint which transcription factor could potentially function together with ENAP1 in seed germination in response to ABA, we re-analyzed publicly available transcriptome data [38,39] and found *ABI5* transcripts were increased most under ABA treatment in time series, and were also most enriched during seed germination (Fig 2F and S2B Fig). We also found that the ABI5 binding motif had a high similarity with YGMCACGTGTC motif (Fig 2G). Importantly, we found about 54% (263/485) of ENAP1 and ABA positively regulated genes had at least one ABI5 binding motif in 1k bp upstream of their TSSs (S2C Fig). Overall, these data strongly suggest that ENAP1 is involved in ABA potentially by functioning together with ABI5.

### ENAP1 interacts with ABI5 both *in vivo* and *in vitro*

**Fig 3:**
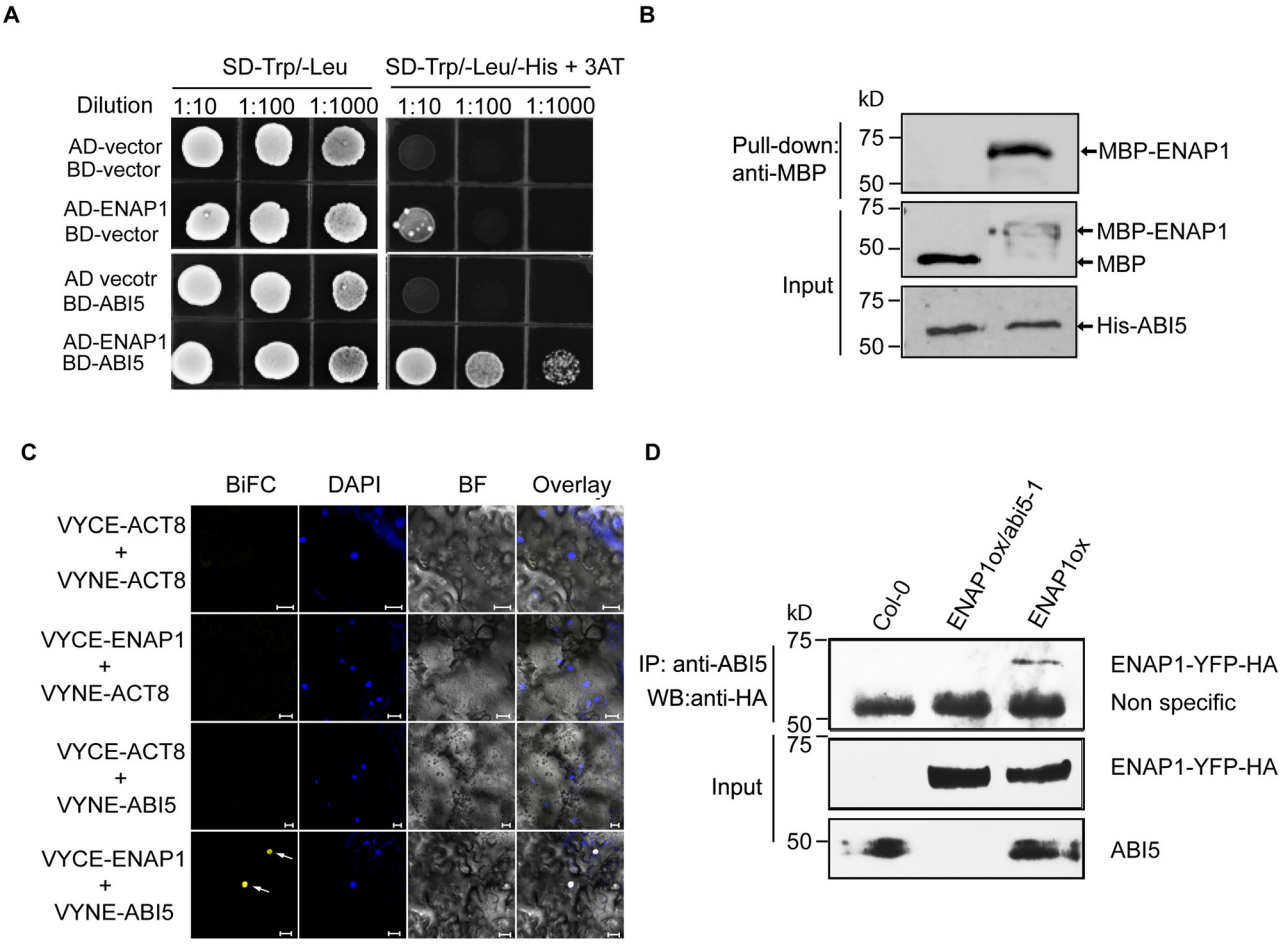
ENAP1 interacts with ABI5 both *in vitro* and *in vivo*. (**A**) Yeast two-hybrid assay to show the interaction between ENAP1 and EIN5. The indicated constructs were co-transformed into yeast. Left panel: yeasts were grown on two-dropout medium as a control; right panel: yeast grown on selective three-dropout medium to evaluate the interaction between ABI5 and ENAP1. (**B**) *In vitro* pull down showing the interaction between ENAP1 and ABI5. The recombinant proteins His-ABI5 and MBP-ENAP1 expressed and purified from *E*.*Coli* were used for the *in vitro* pulldown, and MBP protein served as the control. (**C**) BiFC assay showing that ENAP1 interacts with ABI5. The N-terminus and C-terminus of “Venus” YFP were fused with ABI5 and ENAP1. ACT8 fused with both terminuses were used as negative controls. The fluorescence was observed in the tobacco leaves two days after Agrobacteria infiltration with confocal microscopy. Bars represent 50μm. (**D**) *In vivo* immunoprecipitation assay to demonstrate the interaction of ENAP1 and ABI5. Total proteins extracted from 24h germinated seeds of *ENAP1ox*, *Col-0 and ENAP1ox/abi5-1* with 2μM ABA treatment were immunoprecipitated by using anti-ABI5 antibody. The input served as the loading control. Blots were probed with anti-HA and anti-ABI5 antibodies.

To test our speculation that ENAP1 functions together with ABI5, we first performed yeast two-hybrid assay to examine their interaction. A strong interaction between ENAP1 and ABI5 was detected (Fig 3A). However, no interactions between ENAP1 and other ABFs were detected in the yeast two-hybrid assays, indicating the specificity of ENAP1-ABI5 interaction (S3 Fig). To further validate their interaction, we conducted *in vitro* pulldown assay using recombinant proteins purified from *E*.*coli*. and BiFC assay in tobacco leaves. A strong interaction between ENAP1 and ABI5 was detected in both assays (Fig 3B and 3C). Next, we investigated the interaction between ENAP1 and ABI5 by *in vivo* co-immunoprecipitation. The immunoprecipitation was conducted using the extracts from the seeds germinated with the presence of ABA for 24h, when both ENAP1 and ABI5 were abundantly expressed. Indeed, ENAP1 can be immunoprecipitated by ABI5 in the cell lysis of *ENAP1ox*, but not in the cell lysis of Col-0 or *ENAP1ox/abi5-1* (Fig 3D), showing that ENAP1 interacts with ABI5 both *in vitro* and *in vivo*, providing additional evidence that ENAP1 is involved in ABA signaling via ABI5.

### ENAP1 involves in ABA- repressed seed germination through ABI5

**Fig 4:**
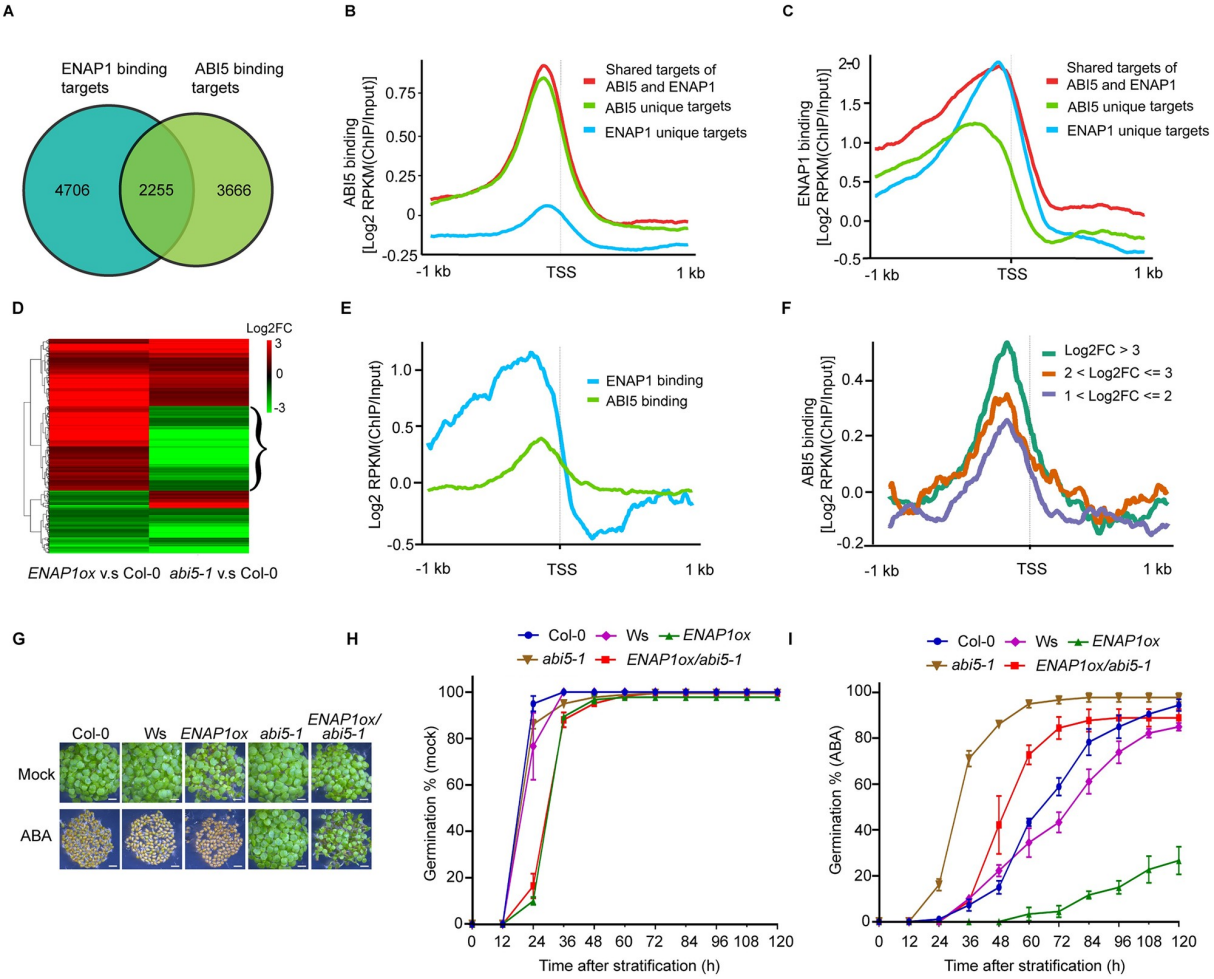
ENAP1 involves in ABA- mediated inhibition of seed germination through ABI5. (**A**) Venn diagram comparing the number of ABI5 and ENAP1 binding genes identified from ChIP-seq. (**B**) Mean enrichment of ABI5 ChIP-seq signal (Log_2_[RPKM(ChIP/Input)]) 1kb up- and down- stream of TSS in genes that are bound by ABI5, ENAP1 or co-bound by ABI5 and ENAP1. (**C**) Mean enrichment of ENAP1 ChIP-seq signal (Log_2_[RPKM(ChIP/Input)]) 1kb up- and down- stream of TSS in genes that are bound by ABI5, ENAP1 or co-bound by ABI5 and ENAP1. (**D**) Heatmap to show the expression pattern of differentially regulated genes in *ENAP1ox* and *abi5-1*. (**E**) Mean enrichment of ABI5 and ENAP1 ChIP-seq signals 1kb up- and down- stream of TSS in genes that are up-regulated in *ENAP1ox* and down-regulated in *abi5-1* (indicated by the Curly bracket in (**D**)). (**F**) Mean enrichment of ABI5 ChIP-seq signals 1kb up- and down- stream of TSS of genes up-regulated in *ENAP1ox* and down-regulated in *abi5-1*. Genes were grouped based on log2(fold change) in *ENAP1ox* v.s. Col-0. (**G**) Photographs of germinating seeds of indicated genotypes at 96h after stratification with or without the presence of 2μM ABA. Bar, 0.2mm. (**H and I**) Germination percentage of seeds of indicated genotypes in the presence of mock (**H**) or 2μM ABA (**I**). Germinated seeds were counted every 12h until 120h after stratification. Each data point represents mean ± s.d. of at least 180 seeds in three replicates.

On the basis of the interaction between ENAP1 and ABI5, we speculated that ENAP1 and ABI5 may co-bind to a cluster of genes to regulate their expression. To test this hypothesis, we re-analyzed the ChIP-seq data of ENAP1 and ABI5 and compared their binding properties [31,44]. Using stringent peak-calling criteria, we found about 38% (2255/5921) of ABI5 bound genes were also bound by ENAP1 (Fig 4A). Heatmap showed that ABI5 and ENAP1 both peaked surrounding the TSS regions of their co-targeted genes (S4A Fig). GO analysis showed that the term “response to ABA stimulus” was overrepresented, further suggesting that ENAP1 is involved in ABA pathway (S4B Fig). Given that ENAP1 interacts with ABI5, we then ask whether the interaction enhances their ability to access the chromatin. To address this question, we compared ABI5 ChIP signals from the genes only bound by ABI5, the genes only bound by ENAP1, or the genes bound both by ENAP1 and ABI5, respectively. Results showed that ABI5 and ENAP1 displayed stronger ChIP signals to their unique target genes at TSS region than genes that are not their targets (Fig 4B and 4C). Notably, ABI5 preferred to bind to the genes that were co-targeted by ABI5 and ENAP1 (Fig 4B). Consistently, ENAP1 showed higher ability to access the genes that are co-targeted by ABI5 and ENAP1 than genes uniquely targeted by ENAP1 (Fig 4C).

We next asked whether ABI5 and ENAP1 collaborate to regulate gene transcription. By re-analyzing the published RNA-seq data in the *abi5-1* seeds, we extracted 282 genes that were up- regulated in *ENAP1ox* and down- regulated in *abi5-1* (indicated by curly bracket in Fig 4D). ABI5 and ENAP1 showed similar binding profiles on those genes (Fig 4E). We divided the 282 genes into three groups according to the expression of log2 fold change in *ENAP1ox* compared to Col-0, and then examined ABI5 binding profiles on the genes in these three groups. We found that ABI5 binding was positively correlated with the levels of the gene expression (Fig 4F), further supporting the conclusion that ENAP1 interacts with ABI5 to co-target ABA-responsive genes to synergistically regulate their expression. To validate the hypothesis in the genetic level, we introduced *abi5-1* mutant into *ENAP1ox* to generate *ENAP1ox/abi5-1* plants and western-blot assay showed that ENAP1 protein levels were not altered by *abi5-1* mutation during seed germination (S4C Fig). In the absence of ABA, *ENAP1ox* as well as *ENAP1ox/abi5-1* seeds displayed delayed germination compared to wild type seeds (Fig 4G and 4H). However, in the presence of ABA, the late seed germination phenotype of *ENAP1ox* was rescued in *ENAP1ox/aib5*, of which the phenotype was more similar to that of *abi5-1* (Fig 4G and 4I). Together, all the data strongly suggest that ENAP1 regulates seed germination through ABI5 in response to ABA.

### ENAP1 depends on ABI5 to deposit H3K9Ac during seed germination

**Fig 5:**
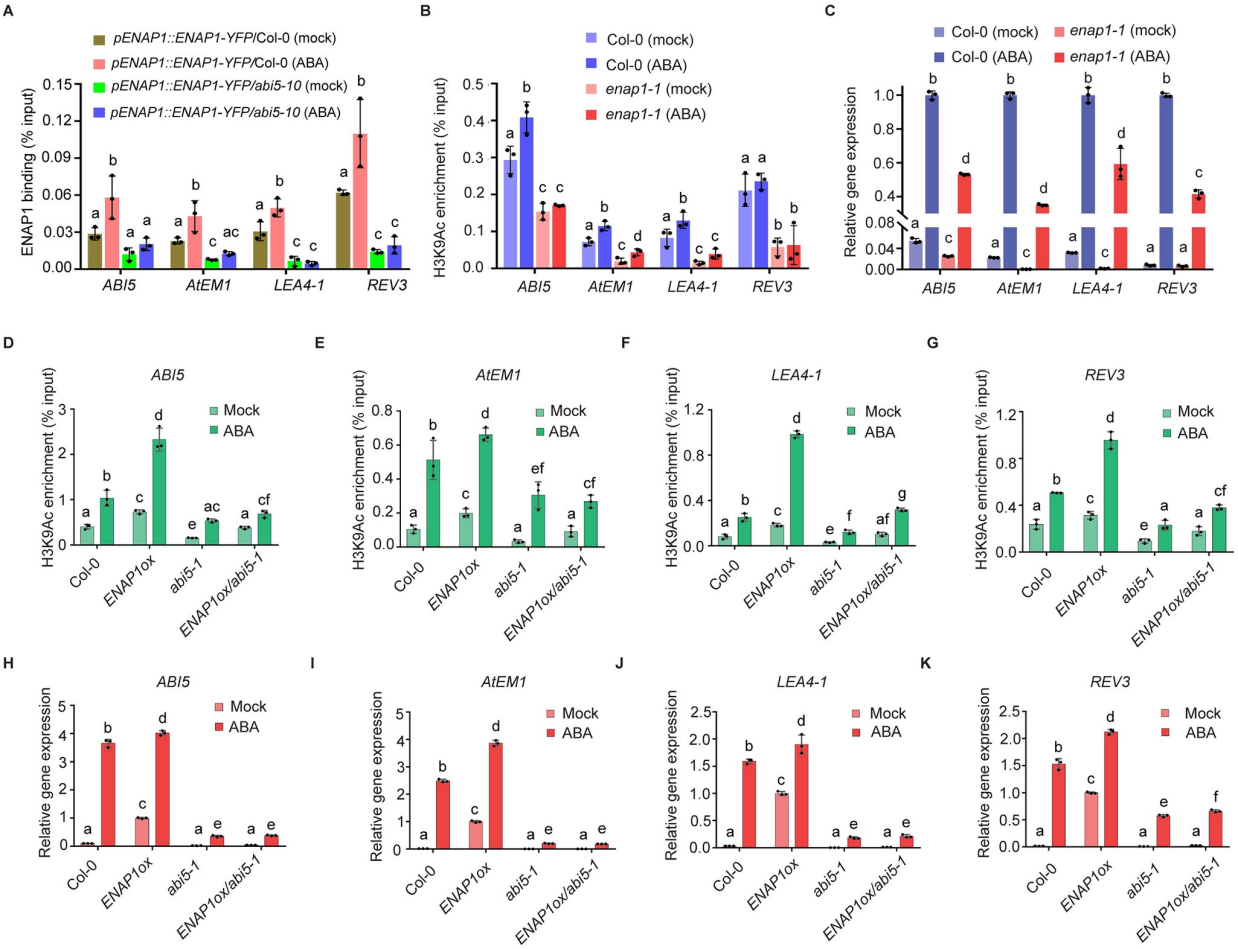
ENAP1 depends on ABI5 to regulate H3K9Ac in response to ABA during seed germination. (**A**) ChIP-qPCR to show ENAP1 binding to the promoters of four representative ENAP1 and ABI5 co-targeted genes. 24h germinated seeds of indicated plants under mock and 2uM ABA treatment were used for the ChIP assay. (**B**) ChIP-qPCR to examine H3K9Ac enrichments in the promoters of the target genes. Col-0 and *enap1-1* seeds that were germinated for 24h in the presence of mock or 0.5μM ABA were used for the ChIP assay. (**C**) qRT-PCR to show target genes’ expression in seeds of Col-0 and *enap1-1*. Total RNAs were extracted from seeds treated as described in (**B**). (**D-G**) ChIP-qPCR to examine H3K9Ac enrichment in the promoters of *ABI5* (**D**), *AtEM1* (**E**), *LEA4-1* (**F**) and *REV3* (**G**). ChIP assay was performed with anti-H3K9Ac antibody in Col-0, *ENAP1ox*, *abi5-1* and *ENAP1ox/abi5* seeds that were germinated for 24h with mock or 2μM ABA treatment. (**H-K**) qRT-PCR analysis of the relative expression of *ABI5* (**H**), *AtEM1* (**I**), *LEA4-1* (**J**) and *REV3* (**K**). Total RNAs were extracted from seeds treated as in (**D**). All data represent means ± s.d. of three replicates. Different letters represent significant differences with *P* < 0.05 in the one-way ANOVA test.

Since the hypersensitivity to ABA in *ENAP1ox* during seed germination is recovered by the *abi5* null mutation (Fig 4G–4I), we next asked how ENAP1 mediated seed germination in response to ABA relies on ABI5 at the molecular level. To address this question, we first examined whether the binding of ENAP1 to the target genes was ABI5 dependent. ChIP-qPCR assays of four representative ENAP1 and ABI5 co-targeted genes, *ABI5*, *AtEM1*, *LEA4-1* and *REV3*, showed that ABI5 was required to enhance the binding of ENAP1 (Fig 5A and S5A–S5E Fig). Given the fact that ENAP1 affects H3Ac in ethylene response [31], we then examined H3Ac enrichment in the promoter regions of those target genes in 24h germinated Col-0 and *ENAP1ox* seeds. We found that the enrichment of H3Ac was much higher in *ENAP1ox* than that in Col-0 (S6A Fig). To define which acetylation species is contributing to the elevation of H3Ac in *ENAP1ox* seeds, we compared the levels of H3K9Ac, H3K14Ac, H3K18Acand H3K27Ac in the promoters of those target genes in 24h germinated seeds. We found that H3K9Ac, but not the other acetylation species, was elevated in *ENAP1ox* (S6B–S6E Fig). Further ChIP-qPCR assays showed that in the presence of ABA, H3K9Ac levels were increased in the promoters of those target genes in Col-0; however, the elevations of H3K9Ac by ABA were largely compromised in *enap1-1* (Fig 5B). Moreover, the expression of those target genes was positively correlated with the changes of H3K9Ac in *enap1-1* seeds under mock and ABA conditions (Fig 5C), showing that ENAP1 promotes histone acetylation and thereby activates gene expression during seed germination.

Finally, we compared the H3K9Ac levels in the promoters of target genes in the 24h germinated seeds from Col-0, *ENAP1ox*, *abi5-1* and *ENAP1ox/abi5-1* with and without the presence of ABA. Consistently, the H3K9Ac levels were significantly higher in *ENAP1ox* than in Col-0 under the mock condition, and the elevation of H3K9Ac by ABA in *ENAP1ox* was more pronounced than that in Col-0 (Fig 5D–5G). By contrast, the H3K9Ac levels in *abi5-1* as well as in *ENAP1ox/abi5-1* were relatively lower than in Col-0 in the absence of ABA (Fig 5D–5G). The elevation of H3K9Ac by ABA was significantly reduced in *abi5-1*. Notably, the elevation of H3K9Ac by ABA in *ENAP1ox* was largely impaired in *ENAP1ox/abi5-1* (Fig 5D–5G). qRT-PCR assays further showed that the expression of the target genes was positively correlated with the H3K9Ac levels in the seeds of above genotypes (Fig 5H–5K). Altogether, these data and the trends that come with them demonstrate that ENAP1 responds to ABA by elevating H3K9Ac in an ABI5 dependent manner, resulting in the upregulation of target gene expression to inhibit seed germination.

### ENAP1 is involved in the positive feedback regulation of *ABI5*

**Fig 6:**
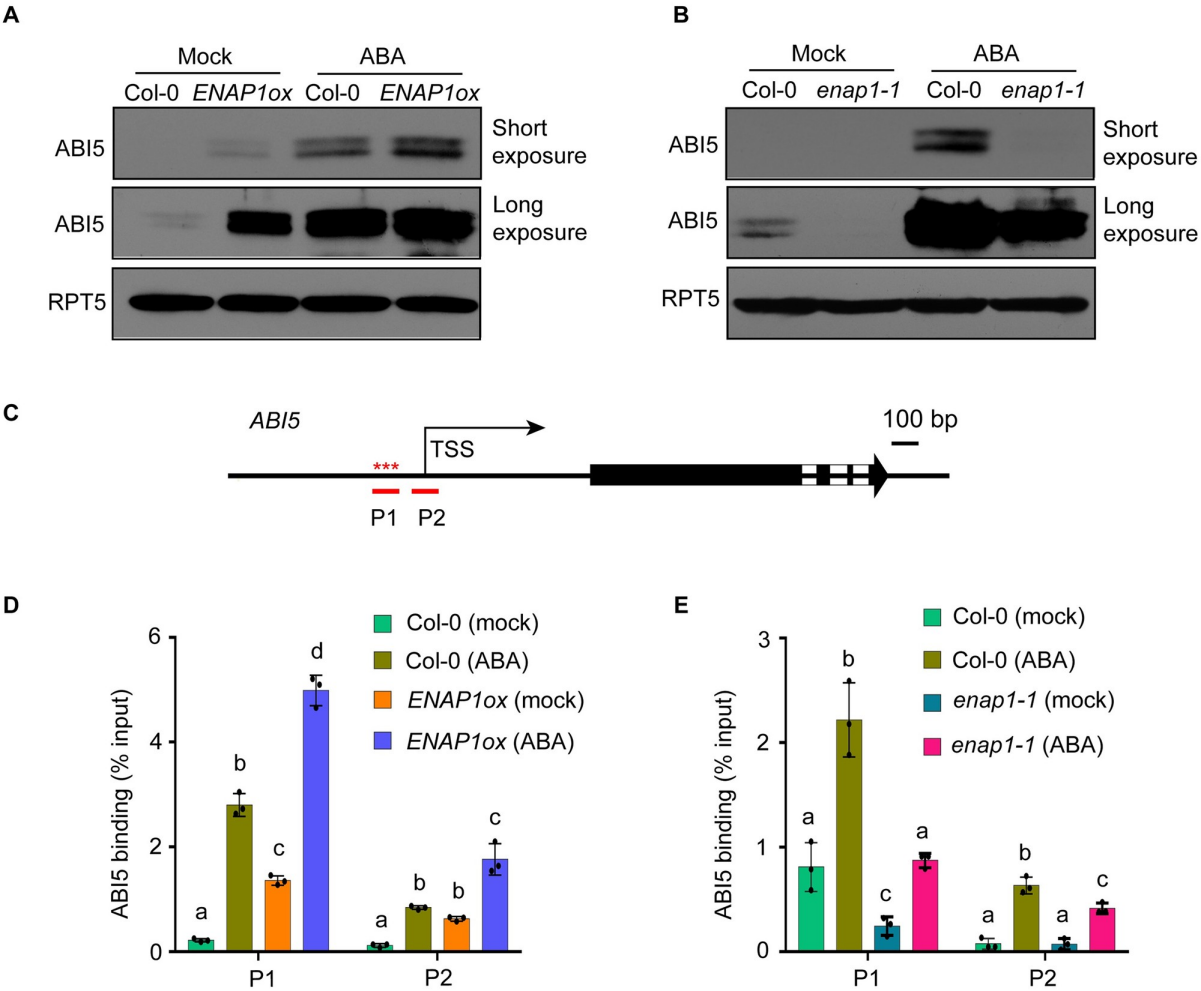
ENAP1 is involved in the positive feedback regulation of *ABI5*. (**A**) Western blot to examine ABI5 protein levels in Col-0 and *ENAP1ox*. Total proteins were extracted from seeds germinated for 24h with the presence of mock or 2μM ABA treatment. RPT5 was used as loading control. (**B**) ABI5 protein levels in Col-0 and *enap1-1*. Total proteins were extracted from seeds germinated for 24h with the presence of mock or 0.5μM ABA treatment. (**C**) Primer locations in the promoter region of *ABI5*. Two primers (P1 and P2) were designed along ABI5 promoter for following ChIP-qPCR. Asterisks represent ABI5 binding motifs. (**D**) ChIP-qPCR to show ABI5 protein enrichment on *ABI5* gene. ChIP assay was performed in seeds treated as in (**A**). (**E**) ABI5 protein enrichment on *ABI5* gene in Col-0 and *enap1-1*. Col-0 and *enap1-1* seeds treated as in (**B**) were used for the ChIP assay. All quantified data represent means ± s.d. of three replicates. Different letters represent significant differences with *P* < 0.05 in the one-way ANOVA test.

As observed in previous studies [14,15], we noticed that ABI5 targets its own gene promoter (S5A and S5C Fig), suggesting that an autoregulation of ABI5 is involved. Additionally, we found that the gene expression of *ABI5* is positively correlated with the levels of *ENAP1* expression (Fig 5C and 5H). Thus, we speculate that the ENAP1- mediated regulation of H3K9Ac is important for recruiting more ABI5 to achieve the positive feedback regulation. To test this idea, we first examined the ABI5 protein levels in both the *enap1-1* and the *ENAP1ox* seeds with or without ABA treatment. Compared to Col-0, the ABI5 proteins were reduced in the *enap1-1* mutant and were elevated in *ENAP1ox* both with and without ABA treatments (Fig 6A and 6B), which is consistent with its transcription changes (Fig 5C and 5H), showing that ENAP1 regulates *ABI5* gene expression in a positive manner. To further examine how ENAP1 regulates ABI5 binding to its own promoter, we scanned the 1k bp upstream of TSS of *ABI5* gene with typical ABI5 motif using FIMO, and identified three ABI5 motifs with a p-value < 0.01 as previously described [15,48] (indicated by asterisks in Fig 6C). Primers (P1 and P2) were designed to profile ABI5 binding over its own promoter (Fig 6C). Accordingly, ABI5 preferred to bind to the P1 region where ABI5 binding motifs were located, and its binding was significantly reinforced in *ENAP1ox* and reduced in the *enap1-1* mutant compared to Col-0 in the absence of ABA. The elevation of ABA- induced ABI5 binding was enhanced in *ENAP1ox* seeds, but significantly decreased in *enap1-1* compared to Col-0 (Fig 6D and 6E). More importantly, the ABI5 binding activity was positively correlated with the levels of *ABI5* gene expression and protein abundance (Figs 5C and 5H and 6), providing profound evidence that ENAP1 assists ABI5 to target its own promoter. We also observed that ABI5 preferred binding to the targets that were also bound by ENAP1 at genome wide, showing the requirement of ENAP1 in the ABI5-mediated positive feedback regulation (Fig 4B). Taken all together, these data demonstrate that the ENAP1-mediated regulation of H3K9Ac is required for ABI5 to achieve the positive feedback regulation and thus promotes *ABI5* dependent transcriptional regulation, leading to an inhibition in seed germination.

## Discussion

**Fig 7:**
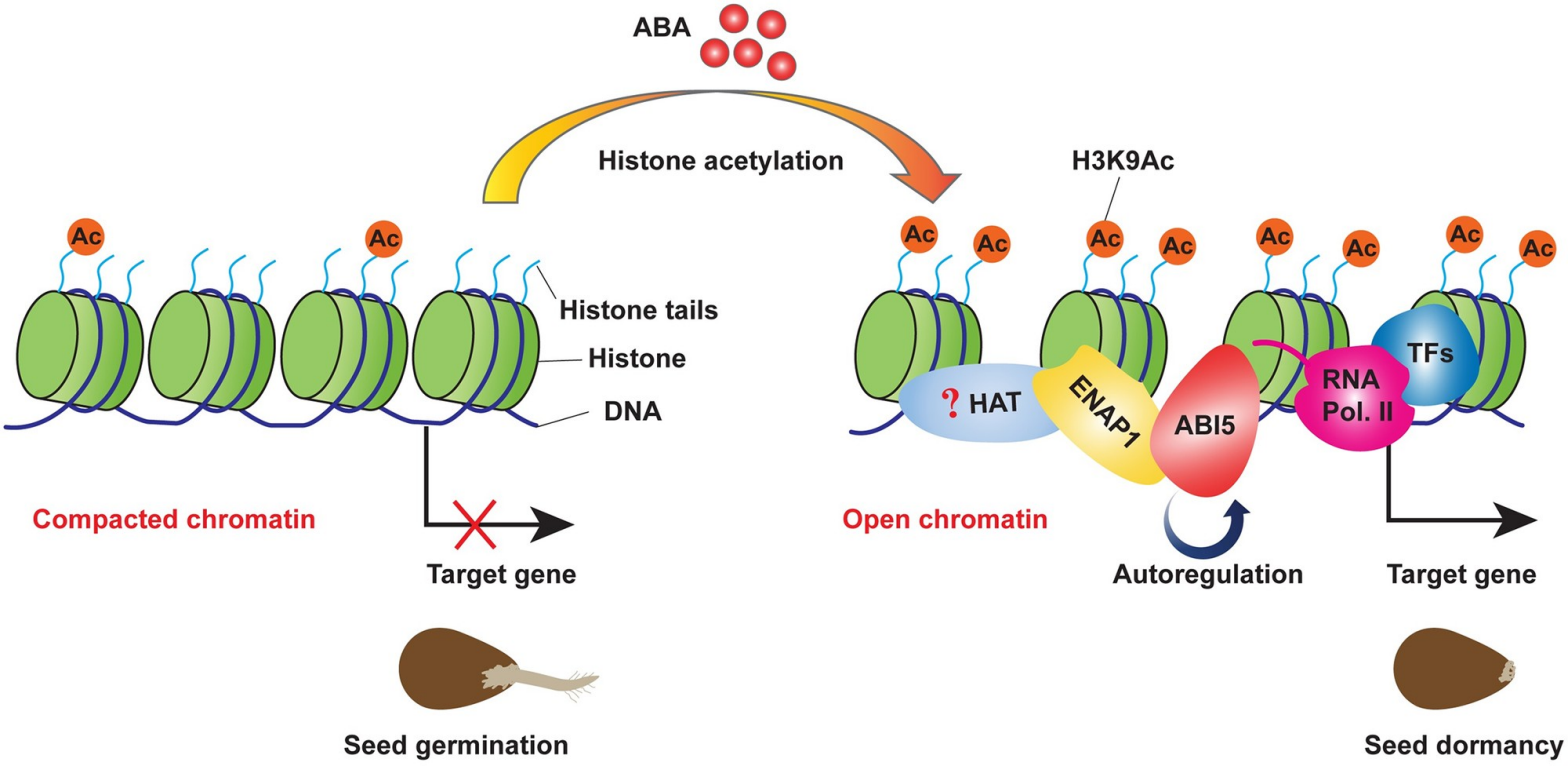
A model to illustrate how ENAP1 inhibits seed germination via H3K9Ac-mediated positive feedback regulation of *ABI5*. ENAP1 serves as a chromatin seat taker to keep a higher level of H3K9Ac, leading to a permissive chromatin status for target genes transcription. When there is a higher level of ABA, accumulated ABI5 prefers binding to the loci where ENAP1 binds with a higher level of H3K9Ac, and the interaction between ENAP1 and ABI5 in turn promotes the elevation of H3K9Ac and recruitment of ABI5, leading to an ABI5-dependent positive feedback regulation of the target genes including *ABI5* itself, resulting in an inhibition of seed germination.

Control of germination timing is one of the most important strategies for flowering plants to optimize their fitness in the natural environment. At the level of individual seed, mechanisms exist to maintain or break dormancy to ensure optimal germinating time in response to environmental stimuli [49]. ABA is one of the most important phytohormones that influences seed dormancy and germination. Given the decrease in ABA content during seed germination and the positive role of ABI5 in ABA signaling, ABI5 plays a critical role in germination as the maintainer of the ABA signal. Many studies have revealed the regulation of ABI5 at transcriptional and post-translational levels, yet epigenetic regulation as well as autoregulation of ABI5 is largely unexplored [9,50–52]. In this study, we provide multiple lines of compelling evidence showing that histone binding protein ENAP1 inhibits seed germination via H3K9Ac mediated positive feedback regulation of ABI5. First of all, genetically, we showed that *ENAP1* gain-of-function (*ENAP1ox*) displayed enhanced ABA sensitivity while deficient mutants exhibited reduced ABA sensitivity in seed germination (Fig 1), and the null mutation of *ABI5* restored such phenotype in *ENAP1ox* (Fig 4H–4I). Secondly, we found that ENAP1 directly interacted with ABI5, but not with other ABFs, both *in vivo* and *in vitro* (Fig 3 and S3 Fig). Thirdly, we found that ENAP1 relies on ABI5 to bind and regulate target genes (Fig 5A and 5H–5K). More importantly, we found that ABI5 and ENAP1 co-target the *ABI5* gene promoter, and ENAP1 regulates *ABI5* gene expression and thereafter protein translation, resulted from the assistance of ENAP1 on ABI5 auto-regulation (Figs 5C and 5H and 6 and S5A–S5C Fig). However, ENAP1 protein is not regulated by ABI5 (S4C Fig). Finally, we found that the positive role of *ENAP1* to regulate H3K9Ac as well as target gene expression was dependent on ABI5 (Fig 5D–5K). Altogether we proposed a model that at the beginning of seed germination when there is a high level of ABA, the histone binding protein ENAP1 elevates H3K9Ac, creating a relax status in the target loci, leading to an enhanced binding and transcription activity of ABI5 to its targets including ABI5 itself, resulting in an inhibition of seed germination (Fig 7).

Histone acetylation was previously shown to be very important in seed germination. For example, HDA6 and HDA19, involving the removal of H3K14Ac and H3K9/18Ac, act redundantly to repress embryonic genes expression after germination, and seed germination of their deficient mutants show hypersensitivity to ABA [21–23]. HDA15 enables deacetylation of H3 and H4, consequently resulting in the repression of ROP genes as well as seed germination [27]. It is now well established that many effects exerted by TFs in eukaryotes are mediated through interactions with a host of coregulators that modify the chromatin state, resulting in a more open (in case of activation) or closed conformation (in case of repression). In this study, we found that H3Ac levels are significantly higher in *ENAP1ox* than that in Col-0 in 24h germinated seeds (S6A Fig). Intriguingly, unlike in ethylene response that H3K14Ac and H3K23Ac are elevated [31], H3K9Ac is significantly regulated during seed germination (S6B–S6E Fig). Most importantly, ABI5 is required for the ENAP1-mediated elevation in H3K9Ac (Fig 5D–5G), which shows that ABI5 plays an important role in regulating transcription by directing the recruitment of the accessory factors, which in this case is the histone binding protein ENAP1. Further studies to identify the HAT or HDAC that associate with AIB5 directly or indirectly will provide more insight into how histone acetylation controls seed germination.

Notably, the levels of H3K9Ac and target gene expression are still slightly elevated in the presence of ABA even in *abi5-1* mutant (Fig 5D–5K), which suggests that there are potential additional ABI5 independent routes to regulate H3K9Ac in the presence of ABA. Additionally, in our study, we focused on H3K9Ac, but whether and how other histone modifications are involved in seed germination are unknown. Further studies on the molecular mechanism of how specific histone H3 loci are determined for acetylation, and what other histone modifications are involved, and how the combinatorial regulation of those histone modifications integrates in the gene expression during seed germination are of interest.

Our previous study showed that ENAP1 was involved in the ethylene response by regulating histone acetylation [31,33]. Moreover, we also proposed that ENAP1 binds chromatin to maintain a relaxed state allowing a rapid response to ethylene [33]. The question is how ENAP1 functions in seed germination. We mentioned that ENAP1 binding targets comprise genes that are involved in a broad spectrum of plant activities, including responses to hormones and stresses [33]. It is possible that ENAP1 plays a role as a placeholder to maintain a relaxed state by regulating histone acetylation. In the presence of different cues, accumulated specific transcription factors interact with ENAP1 to rapidly activate transcription for specific responses. In seed germination process, plant hormones ABA and GA play critical roles and ethylene plays a minor role [49,53]. In the beginning of seed germination, ABI5 is accumulated due to a high level of ABA, which offers the opportunity for ENAP1 to interact with ABI5. As expected, our result demonstrated that ENAP1 interact with ABI5 in the presence of ABA, when ABI5 is accumulated (Fig 3). ChIP-seq analysis showed that ENAP1 and ABI5 share a substantial proportion of binding targets and mutually enhance each other’s binding ability, further suggesting that ENAP1 and ABI5 function together to regulate gene expression (Fig 4A–4C). Of note, both ENAP1 and ABI5 bind to the promoter of *ABI5* gene, and ENAP1 also promotes the gene expression of *ABI5* (Fig 5C and 5H and S5A–S5C Fig), indicating that a positive feedback regulation is involved.

## Supporting information

S1 Table Data and Statistical test summary. (XLSX)

S2 Table Primers used in this study. (XLSX)

S1 Fig Generation of *enap1* loss-of-function mutants by artificial RNAi and CRISPR/Cas9. (**A**) qRT-PCR to show transcript levels of *ENAP1* in *ENAP1ox* lines. Total RNA was extracted from seeds germinated on ½ MS for 36h. Data represents mean ± s.d. The expression levels in *ENAP1ox* lines were compared to Col-0 with unpaired two-tailed t-test. **** *P* < 0.0001. (**B**) Germination rates of Col-0 and *ENAP1ox* seeds under different concentrations of ABA. Seeds of Col-0 and *ENAP1ox* #1 were germinated on ½ MS supplemented with ethanol (mock) or 2μM ABA, and the germination rates at 3^rd^ day after stratification were analyzed. Data represents mean ± s.d. of three replicates. At least 60 seeds were used for each replicate. Unpaired two-tailed t-test were used to compare germination rates in *ENAP1ox* to Col-0 under that ABA concentration. **** *P* < 0.0001. (**C**) Relative expression of *ENAP1* in *amiR-ENAP1* knocking- down lines. Total RNAs were isolated from 10d seedlings of two independent lines. (**D and E**) Germination rates of *enap1* knocking- down lines under treatment of mock (**D**) and 2μM ABA (**E**). Germinated seeds were recorded every 12h until 120h after stratification. Data represents mean ± s.d. of three replicates. Each replicate includes at least 60 seeds. (**F**) Schematic diagram of ENAP1 gene and protein. Upper diagram represents *ENAP1* gene and lower diagram represents the protein. Red solid lines in the upper diagram show the deletions in *eanp1-1* and *enap1-2* generated through CRISPR/Cas9. Colored shapes in lower diagram indicate the protein motifs. (**G**) Gel electrophoresis to show the deletions in *enap1*. *enap1-1* has a 146bp deletion and *enap1-2* has a 30bp deletion. (**H**) Sanger sequencing to show the deletions in *enap1-1* and *enap1-2*. (**I**) RT-PCR showing the expression of remaining *ENAP1* in *enap1-1* and *enap1-2*. Total RNAs were extracted from 10d seedlings. UBQ10 was used as a control.
(TIFF)

S2 Fig Characterization of ENAP1 protein changes in response to ABA. (**A**) ENAP1 protein changes during seed germination in response to ABA. Total proteins were extracted from *ENAP1ox* seeds germinated for indicated time under treatment of mock or 2μM ABA. Anti-HA was used to detected ENAP1 proteins, and RPT5 served as loading control. (**B**) Time series transcription changes of TFs associated with top 10 motifs identified by Tomtom motif comparison tool under the treatment of ABA. Total RNAs from 3d old Col-0 seedlings treated by 10μM (±)-ABA or ethanol for indicated time were used for sequencing library construction. (**C**) Distribution of the numbers of genes including ABI5 binding motif. Totally 485 genes up- regulated by ABA and *ENAP1* were performed ABI5 binding motif searching with FIMO software in the 1kb upstream of TSS. 263 genes were found to have at least one ABI5 binding motif with a *P* < 0.01.
(TIFF)

S3 Fig ENAP1 doesn’t interact with ABFs. The indicated constructs were co-transformed into the yeast. Left panel: yeast grown on selective three-dropout medium to test the interaction between ENAP1 and ABFs; right panel: yeasts were grown on two-dropout medium as a control.
(TIFF)

S4 Fig ENAP1 is involved in ABA response. (**A**) Heatmaps to show ENAP1 and ABI5 binding profiles. Regions between 1kb upstream of TSS and 1kb downstream of TTS of ENAP1 and ABI5 co-targeted genes were plotted. (**B**) GO analysis of ENAP1 and ABI5 co-targeted genes. (**C**) Westernblot to show ABI5 and ENAP1 protein level changes during seed germiantion. Stratified seeds of *ENAP1ox* and *ENAP1ox/abi5-1* were germianted for indicated time, and subjected for total protein extraction. Anti-HA and anti-ABI5 were used to detect ENAP1 and ABI5. H3 was used as the loading control.
(TIFF)

S5 Fig Four representative target genes to show ENAP1 and ABI5 bindings. (**A**) IGV to present ENAP1 and ABI5 bindings to the promoter regions of *ABI5*, *AtEM1*, *LEA4-1* and *REV3*. Dashed box showing the binding peaks. Short solid lines indicate primers used in the ChIP-qPCR. Two primers (P1 and P2) were used for *ABI5* in Fig 6D and 6E. (**B and C**) ChIP-qPCR to validate the binding of ENAP1 (**B**) and ABI5 (**C**) to the promoters of representative genes. Genomic DNA was isolated from *pENAP1*::*ENAP1*:*YFP/Col-0* seeds germinated for 24h on ½ MS supplemented with 2μM ABA. IgG was used as a negative control to immunoprecipitate the genomic DNA. Data represents mean ± s.d. of three replicates. ENAP1 or ABI5 enrichments were compared to IgG enrichments with unpaired two-tailed t-test. **** *P* < 0.0001. (**D**) Westernblot to show ABI5 proteins in *abi5-10* mutant. Total proteins were isolated from seeds of Col-0 and *abi5-10* that were germianted for 24h with or without the presence of 2μM ABA. RPT5 was used as the loading control. (**E**) Western blot to show ENAP1 protein levels in *pEANP1*::*ENAP1-YFP/abi5-10*. Total proteins isolated from seeds germinated for 24h under mock or 2μM ABA treatment were probed with anti-GFP. Asterisks indicate non-specific bands. CBB staining served as the loading control.
(TIFF)

S6 Fig *ENAP1* enhances deposition of H3Ac and H3K9Ac to target gene promoters. (**A-E**) ChIP-qPCR to show the enrichments of H3Ac (**A**), H3K9Ac (**B**), H3K14Ac (**C**), H3K18Ac (**D**) and H3K27Ac (**E**) on the promoter regions of *ABI5*, *AtEM1*, *LEA4-1* and *REV3* in seeds of Col-0 and *ENAP1ox* germinated for 24h on ½ MS. Data represents mean ± s.d. of three replicates. Histone acetylation enrichments in *ENAP1ox* were compared to Col-0 with the unpaired two-tailed t-test. *P < 0.05; ** *P* < 0.01; *** *P* < 0.001.
(TIFF)
