# Supplementary figures and images for: ENAP1 retrains seed germination via H3K9 acetylation mediated positive feedback regulation of ABI5
Source: PLoS Genet. 2021 Dec 15;17(12):e1009955. doi: 10.1371/journal.pgen.1009955 (PMC8673607; doi:10.1371/journal.pgen.1009955)

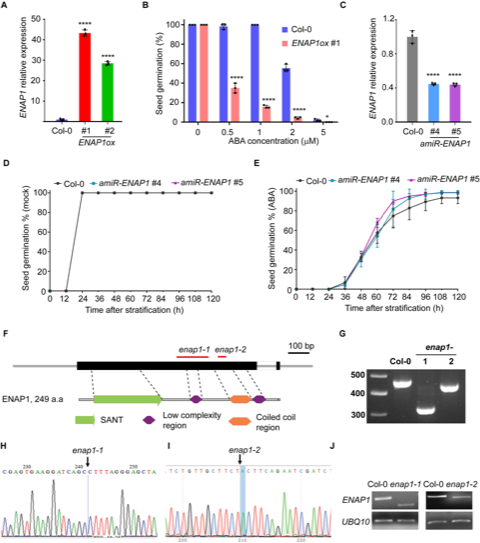

Supplement: S1 Fig — (A) qRT-PCR to show transcript levels of ENAP1 in ENAP1ox lines. Total RNA was extracted from seeds germinated on ½ MS for 36h. Data represents mean ± s.d. The expression levels in ENAP1ox lines were compared to Col-0 with unpaired two-tailed t-test. **** P < 0.0001. (B) Germination rates of Col-0 and ENAP1ox seeds under different concentrations of ABA. Seeds of Col-0 and ENAP1ox #1 were germinated on ½ MS supplemented with ethanol (mock) or 2μM ABA, and the germination rates at 3rd day after stratification were analyzed. Data represents mean ± s.d. of three replicates. At least 60 seeds were used for each replicate. Unpaired two-tailed t-test were used to compare germination rates in ENAP1ox to Col-0 under that ABA concentration. **** P < 0.0001. (C) Relative expression of ENAP1 in amiR-ENAP1 knocking- down lines. Total RNAs were isolated from 10d seedlings of two independent lines. (D and E) Germination rates of enap1 knocking- down lines under treatment of mock (D) and 2μM ABA (E). Germinated seeds were recorded every 12h until 120h after stratification. Data represents mean ± s.d. of three replicates. Each replicate includes at least 60 seeds. (F) Schematic diagram of ENAP1 gene and protein. Upper diagram represents ENAP1 gene and lower diagram represents the protein. Red solid lines in the upper diagram show the deletions in eanp1-1 and enap1-2 generated through CRISPR/Cas9. Colored shapes in lower diagram indicate the protein motifs. (G) Gel electrophoresis to show the deletions in enap1. enap1-1 has a 146bp deletion and enap1-2 has a 30bp deletion. (H) Sanger sequencing to show the deletions in enap1-1 and enap1-2. (I) RT-PCR showing the expression of remaining ENAP1 in enap1-1 and enap1-2. Total RNAs were extracted from 10d seedlings. UBQ10 was used as a control. (TIFF) [file pgen.1009955.s003.tiff]

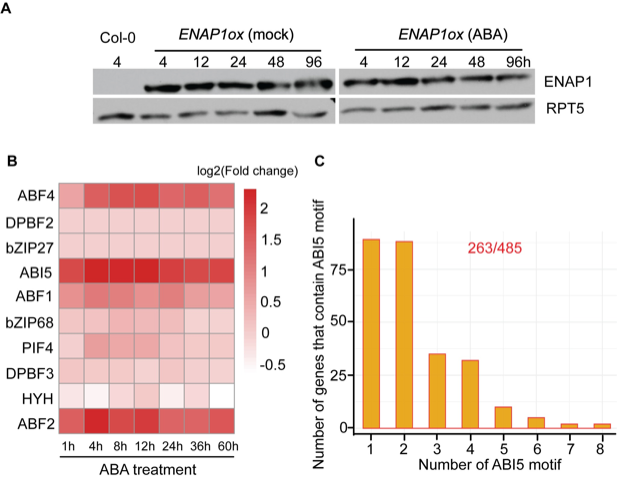

Supplement: S2 Fig — (A) ENAP1 protein changes during seed germination in response to ABA. Total proteins were extracted from ENAP1ox seeds germinated for indicated time under treatment of mock or 2μM ABA. Anti-HA was used to detected ENAP1 proteins, and RPT5 served as loading control. (B) Time series transcription changes of TFs associated with top 10 motifs identified by Tomtom motif comparison tool under the treatment of ABA. Total RNAs from 3d old Col-0 seedlings treated by 10μM (±)-ABA or ethanol for indicated time were used for sequencing library construction. (C) Distribution of the numbers of genes including ABI5 binding motif. Totally 485 genes up- regulated by ABA and ENAP1 were performed ABI5 binding motif searching with FIMO software in the 1kb upstream of TSS. 263 genes were found to have at least one ABI5 binding motif with a P < 0.01. (TIFF) [file pgen.1009955.s004.tiff]

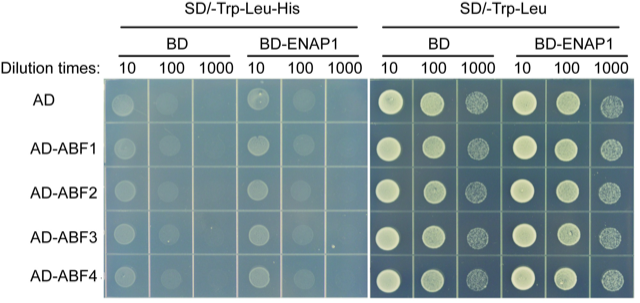

Supplement: S3 Fig — The indicated constructs were co-transformed into the yeast. Left panel: yeast grown on selective three-dropout medium to test the interaction between ENAP1 and ABFs; right panel: yeasts were grown on two-dropout medium as a control. (TIFF) [file pgen.1009955.s005.tiff]

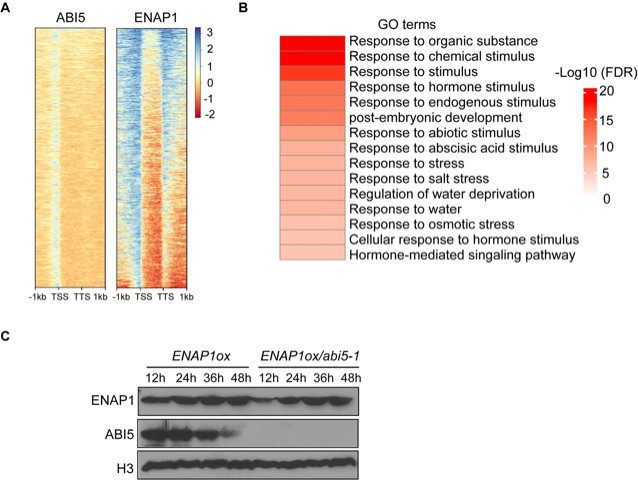

Supplement: S4 Fig — (A) Heatmaps to show ENAP1 and ABI5 binding profiles. Regions between 1kb upstream of TSS and 1kb downstream of TTS of ENAP1 and ABI5 co-targeted genes were plotted. (B) GO analysis of ENAP1 and ABI5 co-targeted genes. (C) Westernblot to show ABI5 and ENAP1 protein level changes during seed germiantion. Stratified seeds of ENAP1ox and ENAP1ox/abi5-1 were germianted for indicated time, and subjected for total protein extraction. Anti-HA and anti-ABI5 were used to detect ENAP1 and ABI5. H3 was used as the loading control. (TIFF) [file pgen.1009955.s006.tiff]

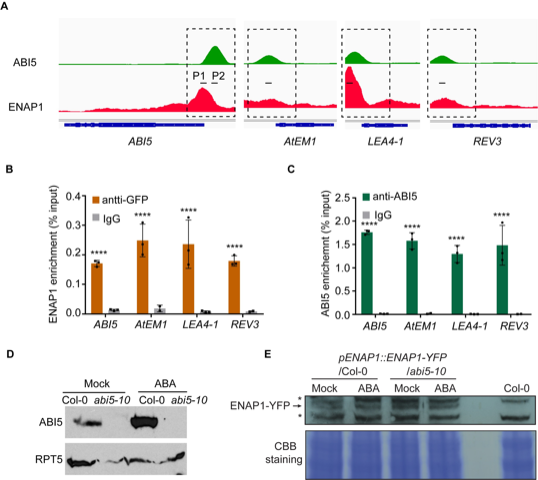

Supplement: S5 Fig — (A) IGV to present ENAP1 and ABI5 bindings to the promoter regions of ABI5, AtEM1, LEA4-1 and REV3. Dashed box showing the binding peaks. Short solid lines indicate primers used in the ChIP-qPCR. Two primers (P1 and P2) were used for ABI5 in Fig 6D and 6E. (B and C) ChIP-qPCR to validate the binding of ENAP1 (B) and ABI5 (C) to the promoters of representative genes. Genomic DNA was isolated from pENAP1::ENAP1:YFP/Col-0 seeds germinated for 24h on ½ MS supplemented with 2μM ABA. IgG was used as a negative control to immunoprecipitate the genomic DNA. Data represents mean ± s.d. of three replicates. ENAP1 or ABI5 enrichments were compared to IgG enrichments with unpaired two-tailed t-test. **** P < 0.0001. (D) Westernblot to show ABI5 proteins in abi5-10 mutant. Total proteins were isolated from seeds of Col-0 and abi5-10 that were germianted for 24h with or without the presence of 2μM ABA. RPT5 was used as the loading control. (E) Western blot to show ENAP1 protein levels in pEANP1::ENAP1-YFP/abi5-10. Total proteins isolated from seeds germinated for 24h under mock or 2μM ABA treatment were probed with anti-GFP. Asterisks indicate non-specific bands. CBB staining served as the loading control. (TIFF) [file pgen.1009955.s007.tiff]

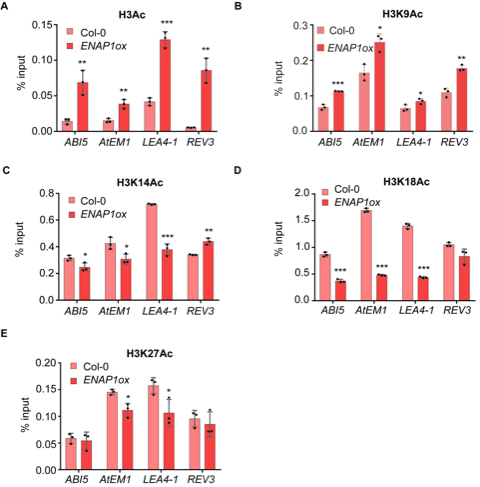

Supplement: S6 Fig — (A-E) ChIP-qPCR to show the enrichments of H3Ac (A), H3K9Ac (B), H3K14Ac (C), H3K18Ac (D) and H3K27Ac (E) on the promoter regions of ABI5, AtEM1, LEA4-1 and REV3 in seeds of Col-0 and ENAP1ox germinated for 24h on ½ MS. Data represents mean ± s.d. of three replicates. Histone acetylation enrichments in ENAP1ox were compared to Col-0 with the unpaired two-tailed t-test. *P < 0.05; ** P < 0.01; *** P < 0.001. (TIFF) [file pgen.1009955.s008.tiff]
